# Organ-differential Roles of Akt/FoxOs Axis as a Key Metabolic Modulator during Aging

**DOI:** 10.14336/AD.2021.0225

**Published:** 2021-10-01

**Authors:** Dae Hyun Kim, EunJin Bang, Sugyeong Ha, Hee Jin Jung, Yeon Ja Choi, Byung Pal Yu, Hae Young Chung

**Affiliations:** ^1^Department of Pharmacy, College of Pharmacy, Pusan National University, Gumjung-gu, Busan 46241, Korea.; ^2^Department of Biopharmaceutical Engineering, Division of Chemistry and Biotechnology, Dongguk University, Gyeongju 38066, Korea.; ^3^Department of Physiology, The University of Texas Health Science Center at San Antonio, TX 78229, USA

**Keywords:** Aging, Akt/FoxOs axis, metabolic organs, non-metabolic organs, inflammation, CR

## Abstract

FoxOs and their post-translational modification by phosphorylation, acetylation, and methylation can affect epigenetic modifications and promote the expression of downstream target genes. Therefore, they ultimately affect cellular and biological functions during aging or occurrence of age-related diseases including cancer, diabetes, and kidney diseases. As known for its key role in aging, FoxOs play various biological roles in the aging process by regulating reactive oxygen species, lipid accumulation, and inflammation. FoxOs regulated by PI3K/Akt pathway modulate the expression of various target genes encoding MnSOD, catalases, PPARγ, and IL-1β during aging, which are associated with age-related diseases. This review highlights the age-dependent differential regulatory mechanism of Akt/FoxOs axis in metabolic and non-metabolic organs. We demonstrated that age-dependent suppression of Akt increases the activity of FoxOs (Akt/FoxOs axis upregulation) in metabolic organs such as liver and muscle. This Akt/FoxOs axis could be modulated and reversed by antiaging paradigm calorie restriction (CR). In contrast, hyperinsulinemia-mediated PI3K/Akt activation inhibited FoxOs activity (Akt/FoxOs axis downregulation) leading to decrease of antioxidant genes expression in non-metabolic organs such as kidneys and lungs during aging. These phenomena are reversed by CR. The results of studies on the process of aging and CR indicate that the Akt/FoxOs axis plays a critical role in regulating metabolic homeostasis, redox stress, and inflammation in various organs during aging process. The benefical actions of CR on the Akt/FoxOs axis in metabolic and non-metabolic organs provide further insights into the molecular mechanisms of organ-differential roles of Akt/FoxOs axis during aging.

Review

## 1. Introduction

Aging causes detrimental changes at the molecular and cellular level that accumulate over time, and ultimately leads to deterioration of tissues and organs, leading to onset of age-related diseases and increased risk of morbidity and mortality [1]. The inflammatory response is associated with age-related diseases such as atherosclerosis, sarcopenia, Alzheimer’s disease, cancer, kidney diseases and fatty liver diseases. Furthermore, many studies have demonstrated that aging is closely related to the phosphatidylinositol-3-kinase (PI3K)/protein kinase B (Akt) signaling pathway [2, 3]. Aging differentially expresses Akt-mediated Forkhead box O (FoxO) levels depending on metabolic- and non-metabolic organs.

The FoxOs protein family consists of FoxO1, FoxO3, FoxO4, and FoxO6, and these are structurally characterized by the presence of a forkhead box DNA binding domain [4]. FoxOs are expressed in ovary, prostate, skeletal muscle, brain, heart, lung, liver, pancreas, spleen, thymus, and testes [5-7]. Key roles of FoxOs transcription factors in induction of downstream target genes involved in regulation of cellular metabolic pathways in the cell cycle, cell death, and oxidative stress response have been reported [8]. Several studies suggest that FoxOs may modulate the aging process for initiation and progression of age-related diseases. However, little information is available on the organ-specific roles of FoxOs and their underlying action mechanisms. Phosphorylation of Akt inactivates FoxOs by inducing their shuttling from the nuclear fraction to the cytoplasm [8-10]. In contrast, suppressed Akt activity leads to elevated nuclear translocation and phosphorylation of FoxOs in aged liver [11]. Increased phosphorylation of FoxOs owing to the activated PI3K/Akt signaling in the aged kidney results in significant changes in insulin levels during the aging process along with other alterations. This observation can be attributed to various functions of FoxOs during aging, which is phosphorylated by Akt activation or inactivation in various aging organs.

Often, the process of aging is associated with many chronic pathological conditions such as vascular diseases, diabetes mellitus, cancer, and metabolic syndrome [12]. The occurrence of diabetes and obesity is associated with insulin resistance [13], which leads to the downregulation of Akt and upregulation of FoxOs, eventually resulting in lipid accumulation in aged liver [11]. FoxOs proteins have various functions in age-associated diseases. For instance, regulation of protein homeostasis during aging progression directly affects the pathogenesis of neuro-degenerative disorders [14-16]. However, protective effects mediated by FoxOs in the process of aging have not been well-documented. It is also unknown whether their signaling pathway and biological effects differ by tissues, disease conditions, or age. FoxOs play a pivotal role in diverse metabolic diseases including obesity, insulin resistance, hyperlipidemia, type 2 diabetes mellitus, and non-alcoholic fatty liver disease (NAFLD) [17]. Most of these conditions are age-related metabolic disorders that are rather associated with dietary factors than aging [18]. The aging process is accompanied by an inflammatory response and metabolic disorders [19]. In addition, insulin resistance is potentially caused by increased secretion of proinflammatory cytokines during aging [20]. Hyperinsulinemia-mediated Akt phosphorylation is increased in aged kidney but decreased in aged liver [21]. Subsequently, FoxOs, a well-known substrate of Akt, becomes suppressed in non-metabolic organs, whereas they are activated in metabolic organ during aging. We summarized the underlying mechanisms responsible for the association of aging with insulin resistance by defining the organ-specific function of the Akt/FoxOs axis in aging.

Calorie restriction (CR) modulates stress responses at cellular as well as physiological levels and extends the lifespan of rodents [22]. Subsequently, many studies conducted in various species have shown that CR modulates the aging progression by regulating the numbers of signaling pathways [23]. Additionally, CR increases genomic stability by reversing DNA methylation changes that occur during aging [24]. The role of FoxOs transcription factors in CR was explored in various organs in the previous reports [25]. The aged mice (24-month-old) demonstrated higher levels of phosphorylated FoxO1 and NF-κB than young mice, and the PI3K/Akt pathway was upregulated during aged kidney. Furthermore, the involvement of FoxO3 in extending the lifespan during CR has been described in a mouse model by Shimokawa *et al*. [26].

This review highlights the importance of the modifications of FoxOs associated with Akt in various organs such as metabolic organs as well as some in non-metabolic organs. In addition, evidence of the role of the Akt/FoxOs axis as a bridge between various organs is presented, and organ-dependent alterations of Akt/FoxOs axis during the aging progression and CR have also been described.

## 2. Differential roles of Akt/FoxOs axis in metabolic- and non-metabolic organs

### 2.1 The function of Akt/FoxOs axis in metabolic organs

FoxO1 is abundantly expressed in metabolic organs such as the liver, white and brown adipose tissues, skeletal muscle, pancreas, and hypothalamus. FoxO1 is a novel regulator of energy metabolism and is highly expressed in the skeletal muscle, which has been identified as a molecular target for insulin signaling modulation [27-29]. FoxO1 promotes glucose production in the liver along with the conversion of carbohydrate oxidation to lipid oxidation in fasting muscle [30]. In the fasting state, hepatic FoxOs [31] are activated owing to the decrease in Akt levels [32]. FoxOs mediates glucose metabolism by converting glucose to acetate for oxidation or to fatty acids [33]. FoxO1 maintains glucose homeostasis by increasing gluconeogenic gene expression in liver, thereby decreasing insulin secretion and insulin sensitivity [34]. Of all the FoxOs family isoforms, FoxO6 is a critical mediator of the production of inflammatory cytokines, such as IL-1β in aged liver [35].

Telomere size and telomerase activity were significantly lower in the FoxO1-KO than those in WT in aged liver [36]. Circulating 17β-estradiol suppressed hepatic glucose production in hepatocytes of mice but failed in Liver-FoxO1-KO mice, suggesting that FoxO1 is required for the inhibition of gluconeogenesis by circulating 17β-estradiols [37]. Additionally, FoxO3-KO mice, although viable, demonstrate infertility due to dysfunctional ovarian follicular development during aging [38] along with dysfunctional muscle regeneration [39]. FoxO3-KO mice downregulate MyoD transcription, a major factor involved in the regulation of myogenesis in myoblasts [39].

The expression of FoxOs inhibits muscle fiber atrophy by inhibiting muscle atrophy F-box (MAFbx)/atrogin-1, muscle RING finger 1 (MuRF1), BNIP3, and cathepsin L mRNA associated with cancer cachexia and sepsis [40]. In addition, the induction of the Mad/Mxd protein facilitates the inhibition of the transcription of Myc target genes, which is required for cell cycle arrest against FoxO3 mediated by the PI3K/Akt signaling pathway [41]. Akt-induced FoxOs phosphorylation leads to nuclear exclusion and prevents atrophy [42]. In addition, Akt/FoxO1, 3/MuRF1 pathway was upregulated in muscle of old mice leading to sarcopenia [43].

FoxO1 suppresses adipogenesis, and FoxO1 haploinsufficiency recovers the numbers and sizes of adipocytes in high-fat diet (HFD)-fed mice [44]. Inhibition of FoxO1 activity ameliorates glucose tolerance and insulin sensitivity and generates energy expenditure under the white adipose tissue of transgenic mice [45]. The inhibition of FoxO1 selectively enhances the expression of PPARγ1 and UCP1 genes that promote oxygen consumption and mitochondrial metabolism in brown adipose tissue. FoxO1 mediates anti-adipogenic actions in response to insulin signaling in the absence of insulin receptor, or insulin receptor substrate, or insulin targeting Akt in cells as well as dysregulated differentiation [46-48]. FoxO1 inhibits apoptosis by cell cycle arrest in the early phase of adipose cell differentiation and terminal differentiation *via* increased expression of cell cycle inhibitor p21. These observations indicate that FoxO1 demonstrates a dual role in both brown and white adipose tissue.

### 2.2 The function of Akt/FoxOs axis in non-metabolic organs

Diabetic nephropathy is associated with the inhibition of FoxOs, NADPH oxidases, and antioxidant enzymes, which contribute to its pathology [49, 50]. FoxO1 also suppresses redox stress, inhibits cell death, and regulates TGF-β signaling in keratinocytes. The role of FoxOs in the wound healing process differs significantly in the *in vivo* diabetic mouse model. FoxO1 exerts beneficial effects in the wound healing process in control mice [51]. FoxO1 modulates wound healing by increasing the expression of TGF-β1 and downstream target genes that are required for keratinocyte migration. In contrast, FoxO1 impairs wound healing in diabetic mice with high levels of oxidative stress. TNFα-driven FoxO1 activity is associated with higher levels of apoptosis and decreased fibroblast proliferation [52]. Moreover, FoxO3 downregulation has also been implicated in the development of hyperplasia in kidney fibroblasts [53]. FoxO1 downregulation increased cellular accumulation of reactive oxygen species (ROS) in response to high levels of glucose in kidney cells [54]. However, increased levels of Beclin-1, Ulk1, Atg4b, Atg9a, and Bnip3 mRNA in the kidney of FoxO3 knockout mice with prolonged occlusion periods have also been demonstrated. The deletion of FoxO3 resulted in a dull autophagy reaction, characterized by lower levels of Atg protein that required for the initiation, nucleation, and elongation of vesicles involved in autophagy [55].

FoxOs are generally activated by relatively small changes in cellular redox levels. These differences are commonly observed in studies using transgenic mice [56]. However, the progression of cellular senescence contributes to aging [57, 58]. As FoxOs are known to be involved in the extension of lifespan, they are expected to reduce senescence. Skurk *et al*. [59] observed that insulin suppresses cardiac muscle atrophy by Akt-dependent inhibition of FoxO3 in the skeletal muscle. In addition, insulin significantly increases Akt phosphorylation in the lung tissue of lean rats, but not in obese mice, indicating that this tissue does not respond to insulin after 12 weeks of HFD [60]. Additionally, Akt expression and activation in the mouse skin increases with age [61]. Smoking inactivates FoxO3, which accelerates the aging of lung tissue during chronic obstructive pulmonary disease [62]. Cells with depletion of FoxO1 exhibited a change in the usage of metabolic substrates from free fatty acids to glucose, which is associated with decreasing lipid accumulation in the heart. Furthermore, keratinocyte-specific FoxO1 deletion downregulates VEGFA gene expression in mucosal and skin wounds, which leads to decreased proliferation of endothelial cell and angiogenesis, re-epithelialization, and dysfunctional granulation [63]. Also, FoxO6 was decreased in aged skin exhibited an increase in melanogenesis, which promote transcription of antioxidant gene that prevented oxidative stress-induced melanogenesis [64].

As shown in Table 1, the Akt/FoxOs axis was upregulated in metabolically active organs including the muscles, liver, and adipose tissue, which was downregulated in metabolic inactive organs including the lungs, kidney, and skin. Furthermore, our results demonstrated that the Akt/FoxOs axis was upregulated in the liver during aging, while it was downregulated in the kidney and lungs in SD rats in an age-dependent manner (unshown data). We previously reported that Akt-induced FoxO1 phosphorylation was reduced in the livers of aged rats, whereas it was increased in the kidney [65]. Different trends of changes in the Akt/FoxOs axis in metabolic and non-metabolic organs during aging are shown in Table 1 [31, 32, 60-62, 66-73].

**Table 1:** Changes in the Akt/FoxOs axis in various organs.

	Tissue	Akt level	Reference	FoxOs level	Reference
Metabolic organs	Adipose	↓	[66, 67]	↑	[68]
	Liver	↓	[32]	↑	[31]
	Muscle	↓	[69]	↑	[70]
Non-metabolic organs	Lung	↑	[60]	↓	[62]
	Kidney	↑	[71]	↓	[72]
	Skin	↑	[61]	↓	[73]

The inhibited Akt activity leads to elevated nuclear activity of FoxOs in metabolic tissue such as the liver and muscle, whereas insulin-mediated Akt activation blunted FoxOs activation in the kidney and lungs during aging. Additionally, reduced growth factor signals activate FoxOs as the Akt-induced FoxOs inhibition axis is disturbed. However, the increase in ROS levels in cells induces the activation of FoxOs *via* specific mechanisms including the c-Jun N-terminal kinase (JNK) pathway [74] and extends the lifespan *via* mitohormesis in muscles [75]. Conversely, FoxOs activation modes do not always lead to the activation of the downstream signaling molecules. For example, Evans-Anderson *et al*. [76] demonstrated that the activation of FoxO4 by ROS and growth factor upregulates the expression of p21 or p27.

FoxO1 inactivation of osteoblasts reduces osteoblast count, bone volume, and the rate of bone formation. In an experimental animal model, the phenotype of osteoblast leading to bone formation in FoxO1-KO mice can be attributed to a suppressed mechanism for antioxidant defense. Elevated ROS activates the p53 signaling pathway leading to cell cycle arrest and limited proliferation of osteoblast cells. N-acetyl cysteine in antioxidative optimal redox levels normalized osteoblast proliferation and bone formation process [77, 78]. The induction of osteoclast formation by parathyroid hormone and IL-1β was followed by an increase in superoxide levels, suggesting that ROS existence is required for osteoclast cell differentiation and resorption of bone in vitro [79]. Both M-CSF and RANKL increase ROS levels and enhance osteoclast formation and activation in osteoclast progenitors [80, 81]. The involvement of redox stress has been indicated in the disease-like bone resorption process associated with estrogen insufficiency [82]. Animal studies with conditional loss or gain of function of FoxOs mutants or mitochondrial catalase in osteoclast cells have demonstrated that FoxOs suppresses the differentiation process in osteoclast cells by stimulating catalase production resulting in the downregulation of H_2_O_2_ levels [83]. In addition, PPARα is activated by MHY908-mediated age-related inflammation *via* modulation of the ROS/Akt/FoxO1 axis in the kidney [84].

In mice and humans, the expression and nuclear localization of FoxO1 and FoxO3 in cartilage decrease at the margins of cartilage exposed to maximum body mass. This may be due to increased secretion of inflammation-inducing cytokines [85]. However, knockdown of FoxO1 and FoxO3 markedly reduced the concentrations of catalase, glutathione peroxidase 1, Sirt1, Beclin-1, and light chain 3 in human articular chondrocytes [86]. The results of this study indicate that aging chondrocyte cells inhibit antioxidants levels and promote susceptibility to cell death associated with ROS [87].

### 2.3 Changes of phenotypes of Akt/FoxOs axis with respect to organ specificity during aging

FoxO1 plays a role in glucose production by insulin *via* metabolic pathways. This process primarily takes place in the liver to promote glucose generation from non-carbohydrate substrates such as glycerol, lactate, and amino acids. As a life-sustaining process, glucose production acts as the sole fuel source for the brain, testes, and erythrocytes during a lengthened fasting period or exercise. Gluconeogenesis primarily occurs in the liver, with small amounts in the kidney [8, 88, 89]. Even though the regulatory role of FoxO1 in the gluconeogenesis-associated gene transcription and expression is widely known, its potential regulatory role in hepatic lipid metabolism is not known.

Age has a significant influence on the clinical characteristics of thyroid dysfunction (TD), *i.e*. hyperthyroidism and hypothyroidism, which cause under-symptoms TD to appear frequently in the elderly [90-93]. As a result, hyperthyroidism or hypothyroidism may be wrongly diagnosed or symptoms may be mistakenly attributed to old age. These results are important for two reasons: i) the prevalence of hyperthyroidism and hypothyroidism increases with age [94, 95], and ii) TD is more likely to generate harmful effects in an aged patient with comorbidities and symptoms including osteoporosis and coronary heart disease [95, 96]. Cardiac aging is characterized by reduced stress tolerance, as the expression of Sur2a, a critical subunit of ATP-sensitive potassium (K_ATP_) channels, decreases with age, resulting in a decrease in the amounts of K_ATP_ channels in the sarcolemma between cardiac myocytes [97]. Additionally, cells that express p16^Ink4a^ are key promoters of such characteristics of age-associated cardiac diseases [98].

**Figure 1.**
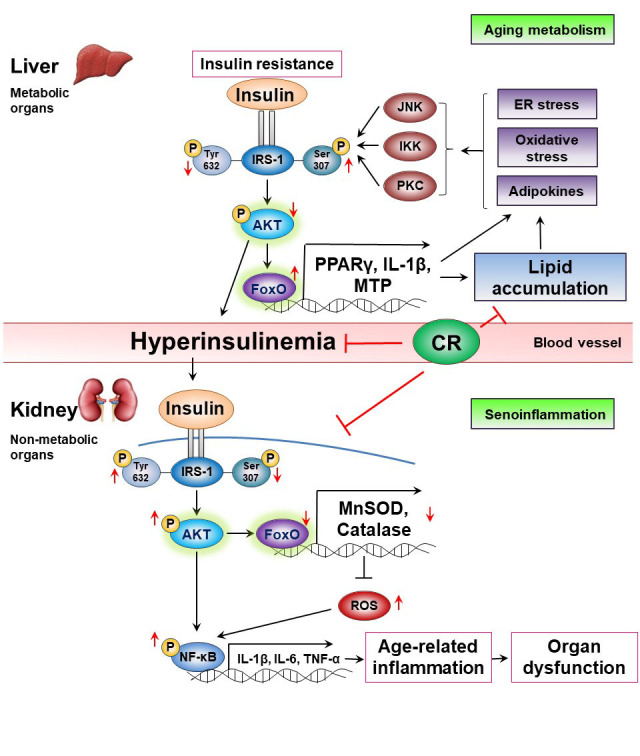
Organ responses based on the Akt/FoxOs axis during aging. Impaired insulin signaling, insulin resistance induces metabolic changes called “aging metabolism” in metabolic organs during aging. The insulin resistance in metabolic organs and tissues such as the liver, muscles, and adipose tissues causes hyperinsulinemia accompanied by Akt inactivation, which increases FoxOs activity (Akt/FoxOs/PPARγ axis upregulation) leading to lipid accumulation. In contrast, the hyperinsulinemia induces Akt activation and inhibits FoxOs activity (Akt/FoxOs/MnSOD axis downregulation) leading to decreases the expression of FoxOs-dependent antioxidant genes such as MnSOD and catalase in non-metabolic organs including the kidneys and the lungs failing to suppress oxidative stress and age-related inflammation. However, CR modulates insulin resistance and hyperinsulinemia, and alleviates age-related inflammation. CR, Calorie restriction; MTP, Microsomal triacylglycerol transfer protein; SOD, Superoxide dismutase; ROS, Reactive oxygen species.

Chronic kidney disease is associated with energy balance, maximum aerobic exercise, as well as tissue glucose uptake [99, 100]. Overnutrition and obesity induce the expression of proinflammatory molecules, such as IL-1β, TNFα, and IL-12, which are related to diverse metabolic diseases [101]. Recently, our research indicated that the downregulation of the expression of enzymes involved in fatty acid oxidation and anti-inflammatory activity of PPARα results in lipid accumulation and renal fibrosis during aging [102]. Indeed, elevated fatty acid can lead to physiological aging [103].

Recent studies have demonstrated that FoxO4 is elevated in aging cells and sustains cell viability by inhibiting p53-mediated cell death. Suppressed interaction of FoxO4 and p53 transcription factors by the designed peptide FoxO4-DRI not only induced p53-mediated cell apoptosis in senescent cells but also promoted fitness, increased amounts of fur, and kidney function in the chronologically aged mouse (*Xpd^TTD/TTD^*) model [104]. Skin changes such as epidermal thinning as well as reduced dermal elasticity and subdermal fat increase the occurrence of stress trauma and skin infection [105].

Aging mediates changes in the digestive process, liver, and endocrine systems in different ways. Liver mass decreases approximately 20-40% during aging, with reduced blood flow [106]. Serum albumin may be degraded, but typically does not change over time in liver chemistry [107]. Aged liver exhibits suppressed synthesis of clotting factor synthesized from vitamin K [108]. Metabolism changes affect the longevity of the experimental animal models, and translational targets can be implemented. Aging is characterized by insulin resistance and suppressed levels of circulating insulin-like growth factor [109]. In addition, aging reduces β-cell regeneration in pancreatic islet cells [110]. Metabolomics methods have distinguished potential longevity characteristics, such as the upregulation of circulating citric acid cycle mediators [111]. Several studies have demonstrated triglycerides (TG) as a nutrient of metabolically active organs that regulated immune and inflammatory effects in adipose tissue [112, 113]. However, inflammatory IL-1β activated by inflammasomes [114] induced lipid accumulation *via* inhibiting PPARα-mediated β-oxidation in the liver [115]. Such insulin resistance conditions stimulate hyperinsulinemia and subsequently activate inflammatory response by inducing Akt signaling pathway in non-metabolic organs such as kidneys during aging (Fig. 1).

Muscle mass and contractility may be suppressed, and muscle mobility may be limited during aging [116]. Age-associated reduction in muscle mass (sarcopenia) is accompanied by reduced muscle quality, as indicated by the infiltration of fat and connective tissue. Inhibition of MuRF1 and MAFbx function is reduced following the inhibition of muscle loss and subsequent attenuation of the pathology associated with muscular atrophy [117]. MuRF1 and MAFbx are expressed during Akt/FoxOs signaling in aged muscle [43]. The data from the current studies demonstrate aging-induced muscle degradation *via* elevation of the Akt/FoxOs axis in muscles.

## 3. Age-related changes and target genes of FoxOs

*Drosophila melanogaster* and *Caenorhabditis elegans* model species have been studied extensively in aging research. FoxOs activation mediates the lifespan extension due to reduced insulin/insulin-like growth factor-like signaling in worms, flies, yeast, and mice. This notable evolutionary preservation has also been observed in humans due to the association of specific genetic variations.

Lack of FoxO3 affects lymph proliferation and inflammation in diverse tissues [118] and is also associated with age-associated infertility [45] and decreased number of neural stem cells [119]. Global deletion of FoxO4 exacerbates colitis in response to inflammatory stimuli [120]. The complete deletion of FoxO6 contributes to memory weakening. The correct synaptic number function regulates gene expression and results in sound neural connectivity [121]. FoxOs can accelerate aging *via* insulin signaling and have been hypothesized to affect longevity by reducing ROS generation and decelerating the extent of redox damage [122]. These findings indicate that FoxOs are involved in the progression of aging and age-associated disorders.

Recent research studies have indicated that FoxOs control a variety of target genes located downstream that are responsible for cell cycle, cell death, and redox stress response [4,8]. One of the major effects of the regulation of FoxOs is phosphorylation by Akt by insulin, growth factors, and its consequential transfer from the nuclear fraction to the cytoplasm [8-10]. FoxO4 appears to suppress cellular oxidative stress levels by directly upregulating gene expression of manganese superoxide dismutase (MnSOD) and catalase in the kidney [123].

Mammalian FoxOs promote hepatic glucose production during the starvation period [9, 124, 125], along with the inhibitory effects of insulin on glucose production in the liver. However, due to the lack of data on FoxOs, little is known about its regulatory role in metabolism or its effect in diabetic conditions. Some studies [126] demonstrated the overexpression of the constitutively active form of FoxO1 in the liver of metabolic organs facilitated the gene expression of lipogenic SREBP-1c and hepatic TG build-up. Furthermore, the regulatory action of FoxO1 was also demonstrated in hepatic lipid metabolism during aging [11]. However, FoxO4 is the critical isoform that acts in FoxOs-mediated transcriptional regulation, which is the ground for skeletal muscle mass reduction in aging diaphragm anabolic syndrome. The Akt activation by the IGF-1 receptor leads to FoxO4 inactivation and the sequential inhibition of the expression of MAFbx/atrogin-1 and MuRF1 genes [127]. Additionally, treatment with β-hydroxy β-methylbutyrate, a metabolite of leucine that modulates muscular atrophy, suppressed dexamethasone-induced muscle wasting by regulating FoxO1 transcription factor and subsequent MuRF1 expression [128].

Available data demonstrate the intricate functions of the FoxOs transcription factors, for instance, under oxidative stress conditions where it not only acts as a positive or negative regulator of cellular function, but also as a cellular modulator of apoptosis, lipogenesis, and inflammation. As summarized in Table 2, accumulated evidence indicates that the FoxOs functions and the expression of their target genes were differential shown according to species and tissues, leading to changes in many physiological phenomena.

**Table 2:** Functions of FoxOs and expression of target genes in various organs.

Organs	Function	Genes
Pancreatic β-cells	Repression of β-cell proliferation	PDX-1, NGN3, NKX61, CyclinD1
	Protection against oxidative stress	MafA, NeuroD
Liver	Increase of gluconeogenesis in mice	G6P, PEPCK, PGC-1a
	Reduced triglyceride levels in pigs, mice	ApoCIII, MTP
Adipose tissue	Control of differentiation	p21, PPARγ
Hypothalamus	Acute orexigenic effect	Agrp, Npy
Brain	Protection against neuronal death	Bim, Fas ligand
Skeletal muscle	Repression of differentiation	Atrogin-1, MuRF1
	Induction of muscle atrophy	MAFbx
Vascular endothelial cells	Regulation of endothelial stability	Ang-2, sprouty2
Smooth muscle cells	Repression of differentiation	Myocardin
Kidney	Protection of lipotoxicity and disease	Bcl-2, Bax, MnSOD, Bim
Testis	Regulation of apoptosis	HOX genes
Heart	Protection of heart against ischemia in mouse	MnSOD, Catalase
	Inhibition of cardiac mass loss in rat	Autophagy genes
Thymus	Regulation of lymphocyte homeostasis	p27
	Control of Treg cell differentiation	FoxP3
Lung	Regulation of lung tumor in mice	p27
	Suppression of lung adenocarcinoma in humans	GADD45

## 4. Modulation of Akt-mediated FoxOs activity by CR for a better understanding of organ based-differential roles of Akt/FoxOs axis

The ROS-mediated regulation of FoxOs may explain its distinct involvement in aging. FoxOs are generally activated by relatively minor alternations in cellular ROS levels and are inactivated with high ROS levels. These changes were clearly observed in transgenic mouse models [56]. Similarly, cellular senescence is a risk factor for aging [57, 58], and activation of FoxOs may extend lifespan through delaying aging process.

Metabolism rates regulate the progression of aging in animals stems from the recognition of the critical link between energy metabolism and homeostasis maintenance. Increased energy expenditure expedites the aging process. It is well established that CR slows down the development of pathologies associated with aging its progression and prolongs lifespan [22, 129-131]. In *C. elegans* and *Drosophila* models, CR increases the lifespan independent of FoxOs regulation [132]. Previous studies on CR have demonstrated its efficacy against the aging progression and have identified several major regulatory signaling pathways [23]. Previous epigenetic studies have indicated that chromosomal or gene promoter regions corresponding to DNA methylation and histone modifications exert functional effects on aging [133]. CR epigenetically modulates the aging process [134] by methods such as mediating an increase in genomic stability by reversing age-associated histone acetylation and alterations in DNA methylation [24]. CR upregulates the expression of certain genes that are involved in cell cycle arrest, such as p21, DNA repair (i.e. Gadd45α), apoptosis (i.e. Bim), and the response to redox stress (i.e. MnSOD) in metabolic organs such as the liver. CR promotes FoxO1 binding in the liver in response to glucose-mediated insulin signal activation [135]. However, Edström *et al*. [136] demonstrated that unlike acute atrophy induced by CR, chronic atrophy caused by diseases, disuse or denervation leads to MAFbx/atrogin-1 and MuRF1 downregulation in the skeletal muscle of 30-month-old rats.

The inhibition of insulin by CR interrupted age-related FoxO6 and FoxO3 reduction by blocking the PI3K/Akt pathway in non-metabolic organs such as the kidney [137, 138]. Subsequently, several studies have supported that CR could slow the aging process in diverse species and regulated several regulatory signaling pathways [23]. Kim *et al*. [25] reported that FoxO1 was activated and NF-κB was inactivated by CR as observed in aged kidney tissues obtained from *ad libitum* fed rats and rats subjected to 40% CR.

In a recent study, the activation of Akt under insulin signaling increased, resulting in an increased inactivation of phosphorylated FoxOs, whereas it was increased by CR in metabolic organs. In contrast, the activation of FoxO1 *via* the inhibition of the PI3K/Akt pathway in non-metabolic organs was suppressed by CR [13, 65, 84, 139]. These results suggest that the Akt/FoxOs axis demonstrates differential actions by organs. This reviews the important findings on the alterations of FoxOs in association with aging and explains its modulation by CR as a potential underlying mechanism affecting the progression of aging.

## 5. Altered Akt/FoxOs signaling pathway in diabetic conditions and aging tissues

In obese patients, excessive lipids accumulation in tissues other than adipose tissue adds to organ damage *via* adipogenic toxicity [140]. This toxic process is complex but can be described by the adipose tissue expandability hypothesis [141-143]. When the capacity of adipose tissue storage is exceeded, the lipid flux increases toward the non-adipose tissues, and lipids begin to accumulate in ectopic sites. Ectopic lipid deposits in different cell types, such as myocytes, hepatocytes, and β-cells, initiate adverse physiological effects such as insulin resistance and apoptosis. Recently, studies have demonstrated that the renal deposits and detrimental effects of lipids may lead to kidney pathology [144, 145]. In particular, saturated fatty acids lead to insulin resistance in podocytes that maintain the integrity of the glomerular filtration barrier in the normal kidney in non-metabolic organ [146], and in proximal tubular cells that lead to cellular dysfunction and cell death *via* apoptosis and necrosis [147].

The association of diabetes with dysfunctional mitochondrial respiratory system in the liver, heart, and kidney of diabetic animals has been demonstrated over 35 years [148-154]. Despite this long history, little is known about mitochondrial dysfunction in diabetics or the mechanisms bridging primary metabolic dysfunction in insulin and blood glucose to metabolic diseases.

Zhang *et al*. [155] demonstrated genetic and physiological evidence indicating that FoxO1 acts as a crucial transcription factor for the IRE as FoxO1 inactivation reduced the transcription of genes encoding gluconeogenic enzymes (PEPCK and G6pc) and suppressed the blood glucose concentrations in the animal model. In contrast, inactivation of FoxO3 promoted the expression of genes encoding lipogenic enzymes, fatty acid synthase, and hydroxy-3-methylglutaryl-CoA reductase. Simultaneous inactivation of both FoxO1 and FoxO3 synergistically induced the expression of lipogenic enzymes including glucokinase (Gck) and further promoted the serum levels of TG, cholesterol, and lipid secretion and that might result in hepatosteatosis. Recently, Dong’s group highlighted the importance of FoxO6 dysregulation in the dual pathogenesis of fasting hyperglycemia and hyperlipidemia in diabetes [156].

Targeting the hepatic insulin/Akt/FoxO1 signaling pathway could be a strategy in impeding the progression of diabetes mellitus. High glucose-induced cell apoptosis in human kidney 2 (HK-2) cells acts by blocking the ROS-responsive Akt/FoxOs signaling pathway in diabetic nephropathy [157]. Several studies have demonstrated that Akt is associated with the expansion of the glomerular matrix [158], apoptosis in podocytes [159], and metastasis of mature tubular epithelial cells by mediating epithelial-to-mesenchymal transition [160]. The Akt kinase exhibits an anti-apoptotic effect in HK-2 cells *via* post-translational regulation of different signaling molecules, where FoxO3 acts as a critical downstream transcription factor [161].

Akt-induced phosphorylation of FoxOs family isoforms, namely, FoxO1 and FoxO3, could promote translocation from the nucleus to the cytoplasm, and consequently inhibit the transcription of downstream target genes such as Bim and Fas-Ligand (Fas-L) that are involved in apoptosis [162]. Decreased phosphorylation of Akt and FoxOs family proteins is related to cellular apoptosis in high glucose-treated kidney cells [163, 164]. However, diabetes nephropathy can inhibit the expression of Fas ligand by increasing FoxO3 phosphorylation and transcriptional inactivation *via* stimulation of the PI3K/Akt pathway [164].

## 6. Discussion

The potential regulatory roles of FoxOs family isoforms during the aging process and the effects of CR on FoxOs activities provide an interesting insight into the participation of FoxOs in the aging process. Inhibition of FoxO1 affects age-associated insulin resistance and energy metabolism, particularly under normal dietary conditions. However, the pivotal role of FoxOs is observed only under CR conditions.

FoxOs are controlled by a variety of growth factors and paracrine hormones, whose activity is securely regulated by post-translational modifications such as phosphorylation, acetylation, methylation, and ubiquitination, as well as physical interactions with different proteins and transcription factors. Additional studies on the post-translational modifications and protein-protein interactions will help elucidate how the FoxOs family isoforms lead to environmental stimuli that mediate the transcription and expression of specific genes and cellular functions to impede age-associated pathological conditions. This paper reviewed the regulatory roles of FoxOs family members during aging to provide strategic insight into potential intervention strategies for the promotion of health.

Numbers of hypotheses have been suggested for aging in past decades, including the molecular inflammation hypothesis. Such a hypothesis involving molecular insulin resistance and inflammation is suggested based on the activity of key FoxOs and its downstream signaling pathway, which plays an important regulatory role in activating systemic inflammation during the aging process upon the induction of adiposity. Chronic inflammation caused by ectopic fat in obese conditions as well as liver and muscle lipid accumulation further deteriorate the insulin resistance conditions. Chronic inflammation prolongs an insulin resistant state, and the association between chronic inflammation and adiposity likely accelerates the aging process. However, redox stress, ER stress, and age-related metabolites impair insulin signaling *via* JNK, IKK, and PKC pathways in metabolically active organs, leading to insulin resistance, hyperinsulinemia, and hyperlipidemia through Akt/FoxOs axis upregulation, which we defined as “aging metabolism or senometabolism” meaning metabolic changes in aging process. The insulin resistance initiates the induction of hyperinsulinemia and enhances the expression of proinflammatory genes encoding for factors such as cytokines and chemokines *via* Akt/FoxOs axis downregulation and NF-κB activation, leading to age-related inflammation (senoinflammation) in non-metabolic organs (Fig. 1). Our previous study confirmed that FoxO6-mediated IL-1β is involved in hepatic inflammation and insulin resistance *via* TF/PAR2/Akt pathway in aging and diabetic liver [35].

The data presented in this review indicate that insulin resistance, change of Akt/FoxOs axis in metabolic organs such as the liver and muscles during aging leads to aging metabolism such as hyperinsulinemia, hyperlipidemia, and age-related metabolic changes. The hyperinsulinemia induces age-related senoinflammation *via* insulin-dependent Akt activation leading to organ dysfunction in non-metabolic organs, namely, the kidneys and lungs. Elucidation of the molecular mechanisms in metabolic organs and non-metabolic organs based on the Akt/FoxOs axis and examination of the regulatory role of CR will provide insights for the development of potential anti-aging interventions.
